# Supplementary material for: Whole Transcriptome Analysis of Intervention Effect of Sophora subprostrate Polysaccharide on Inflammation in PCV2 Infected Murine Splenic Lymphocytes
Source: Curr Issues Mol Biol. 2023 Jul 20;45(7):6067–84. doi: 10.3390/cimb45070383 (PMC10377888; doi:10.3390/cimb45070383)
Supplement: Supplementary file 1 [file cimb-45-00383-s001.zip › cimb-2483591-supplementary.pdf]

Supplementary table S1. Output statistics of the sequencing reads for each sample

Sample	Raw reads	Clean reads	Q20(%)	Q30(%)	GC(%)
C-1	87264494	86855430	97.34	93.02	49.85
C-2	82165908	81768490	97.31	93.05	49.73
C-3	96315314	95884780	97.58	93.51	49.40
V-1	86021402	85619102	97.49	93.41	51.54
V-2	99261282	98804464	97.66	93.73	50.65
V-3	99586532	99153736	97.58	93.57	51.15
SV-1	101535398	101026400	97.60	93.70	51.89
SV-2	87956328	87506042	97.28	93.05	51.09
SV-3	94681750	94220542	97.29	93.10	51.92

Supplementary table S2. Transcription factor targeting analysis

C vs V			V vs SV		
NO.	Genbank	Symbol	NO.	Genbank	Symbol
1	ENSMUSG00000000782	Tcf7	1	ENSMUSG00000008193	Spib
2	ENSMUSG00000015619	Gata3	2	ENSMUSG00000008496	Pou2f2
3	ENSMUSG00000015709	Arnt2	3	ENSMUSG00000009741	Ubp1
4	ENSMUSG00000015846	Rxra	4	ENSMUSG00000018899	Irf1
5	ENSMUSG00000017861	Mybl2	5	ENSMUSG00000018983	E2f2
6	ENSMUSG00000018899	Irf1	6	ENSMUSG00000020167	Tcf3
7	ENSMUSG00000018983	E2f2	7	ENSMUSG00000020644	Id2
8	ENSMUSG00000019982	Myb	8	ENSMUSG00000022286	Grhl2
9	ENSMUSG00000022479	Vdr	9	ENSMUSG00000024431	Nr3c1
10	ENSMUSG00000027490	E2f1	10	ENSMUSG00000026104	Stat1
11	ENSMUSG00000028163	Nfkb1	11	ENSMUSG00000026628	Atf3
12	ENSMUSG00000029238	Clock	12	ENSMUSG00000029014	Dnajc2
13	ENSMUSG00000030256	Bhlhe41	13	ENSMUSG00000032481	Smarcc1
14	ENSMUSG00000032035	Ets1	14	ENSMUSG00000034041	Lyl1

15	ENSMUSG00000034957	Cebpa	15	ENSMUSG00000039191	Rbpj
16	ENSMUSG00000039153	Runx2	16	ENSMUSG00000040187	Arntl2
17	ENSMUSG00000040033	Stat2	17	ENSMUSG00000041540	Sox5
18	ENSMUSG00000042745	Id1	18	ENSMUSG00000048001	Hes5
19	ENSMUSG00000046532	Ar	19	ENSMUSG00000051910	Sox6
20	ENSMUSG00000048251	Bcl11b	20	ENSMUSG00000052271	Bhlha15
21	ENSMUSG00000052684	Jun	21	ENSMUSG00000052684	Jun
			22	ENSMUSG00000063889	Crem

Supplementary table S3. Primer sequence of mRNA

Gene	Primer sequence (5’-3’)	Product length（bp）
CXCL10	F: TCTCTCCATCACTCCCCTTTA<br>R: GCTTCGGCAGTTACTTTTGTC	151
CXCL9	F: TTGTATGTGTGAGAGGTAGAAAGG<br>R: AAAAAGTGGTAAGCAAGCAAGGAG	110
STAT1	F: GCTGCCTATGATGTCTCGTTTG<br>R:GCTTTTCCGTATGTTGTGCTG	123
IRF1	F: GCTGCTGGTCTTGCTGGGTACT<br>R: TGGGGCGATTCAAACTTTTCTT	120
SAMHD1	F: CTTGGCCGTGGTGTCTCTTGA<br>R: CAGTGGGCTGGGATTTTGGTT	249
GBP7	F:ATGCCAGAGGACCAGTTGGA<br>R: CAGAGTCGCCAGTCGGTTT	132
SOCS1	F:CGCTCCTTGGGGTCTGTTG<br>R: TGCGTGCTACCATCCTACTCG	135
IFIT2	F: CCATTGCGAACTACCGTCTG<br>R: CCTCTAACTTCTTCCTATCCCCAC	393
SP140	F: GCCATCACCAAGCCGTTTC<br>R: GTGGGTTGCTGTGCTCCTG	395
FURIN	F: AAGGACATCGGCAAACGG<br>R: TGGTCAGCGTCCCATAGTTG	319
ZBP1	F: AACCCTCAATCAAGTCCTTTACC<br>R: TCTTTGGCTGTCGTCATTCC	231

GBP4	F: TCAAGGCAGGTCAGGGTCTAC<br>R: CTGTTCCAACCAGCAAGATGA	125
GBP2	F: CCCTGAAGCAAAACAAATCG<br>R: CCCCAAAATGGAGTCGCATA	184
Serpina3g	F: GCTGAGACAGGCACAGAGGC<br>R: GCATAGCGGATCACCAAAACA	249
JUN	F: TTCAAAGCTCGGCATCGC<br>R: GGGCATCGTCGTAGAAGGTC	300
RPS2	F: TCATTGATTTCTTCCTGGGTGC<br>R: GCGACGAAAGCCTTGAACC	109
TNF-α	F: CTACTGAACTTCGGGGTGATCG<br>R: CCTCCACTTGGTGGTTTGTGA	112
ACOD1	F: CTTCAGGCTCCCACCGACA<br>R: GGCAGGGCTTCCGATAGAG	141
β-*actin*	F: GCTCTGGCTCCTAGCACCAT<br>R: GCCACCGATCCACACAGAGT	75

Supplementary table S4. Primer sequence of LncRNA

Gene	Primer sequence (5'-3')
MSTRG.17554.2	F: CTCCCTTGCTGAGGCTGTCC<br>R: GGTCCCGTTAGAATGTTGCTTT
MSTRG.4833.1	F: TTTCCACTTCACTGGCTTTGC<br>R: GCCTCACTATCTTGCCCGAC
MSTRG.9203.1	F: AGTGTATTCTTTGGAGCCTTTGC<br>R: GCTGGAGACTGTGGGGATGT
MSTRG.9900.7	F: CCTAGCGAGAAACCTCAGAAATC<br>R: AACAAGACCAAGAACCAAGCAG
MSTRG.12578.2	F: TCCCTGAGCGCCGAGAA<br>R: CCAACCACAGAACCTTTGACCT
MSTRG.4832.1	F: GGATGCCACTTCCACCCTC<br>R: CTGCACCATTCTCCATTCCTACT

Supplementary table S5. Primer sequence of miRNA

Gene	Primer sequence (5'-3')
miR-187-y	GTCTTGTGTTGCAGCCGGA
miR-372-y	TGCTGCGACATTTGAGCGT
miR-125-z	TCCCTGAGACCCTTAACCTGTG
let-7-z	GGCGCTCTGAGGTAGTAGATTGTATAGTT
